# Deep Learning Facilitates Distinguishing Histologic Subtypes of Pulmonary Neuroendocrine Tumors on Digital Whole-Slide Images

**DOI:** 10.3390/cancers14071740

**Published:** 2022-03-29

**Authors:** Marius Ilié, Jonathan Benzaquen, Paul Tourniaire, Simon Heeke, Nicholas Ayache, Hervé Delingette, Elodie Long-Mira, Sandra Lassalle, Marame Hamila, Julien Fayada, Josiane Otto, Charlotte Cohen, Abel Gomez-Caro, Jean-Philippe Berthet, Charles-Hugo Marquette, Véronique Hofman, Christophe Bontoux, Paul Hofman

**Affiliations:** 1 Laboratory of Clinical and Experimental Pathology, Centre Hospitalier Universitaire de Nice, FHU OncoAge, Université Côte d’Azur, 06000 Nice, France; long-mira.e@chu-nice.fr (E.L.-M.); lassalle.s@chu-nice.fr (S.L.); hamila.m@chu-nice.fr (M.H.); hofman.v@chu-nice.fr (V.H.); bontoux.c@chu-nice.fr (C.B.); 2 Hospital-Related Biobank (BB-0033-00025), Centre Hospitalier Universitaire de Nice, FHU OncoAge, Université Côte d’Azur, 06000 Nice, France; fayada.j@chu-nice.fr; 3 Team 4, Institute of Research on Cancer and Aging, CNRS INSERM, Centre Antoine Lacassagne, Université Côte d’Azur, 06100 Nice, France; benzaquen.j@chu-nice.fr (J.B.); berthet.jp@chu-nice.fr (J.-P.B.); marquette.c@chu-nice.fr (C.-H.M.); 4 Department of Pulmonary Medicine and Oncology, Centre Hospitalier Universitaire de Nice, FHU OncoAge, Université Côte d’Azur, 06000 Nice, France; 5 Epione Team, Inria, Université Côte d’Azur, 06220 Sophia Antipolis, France; paul.tourniaire@inria.fr (P.T.); nicholas.ayache@inria.fr (N.A.); herve.delingette@inria.fr (H.D.); 6 Department of Thoracic/Head and Neck Medical Oncology, University of Texas MD Anderson Cancer Center, Houston, TX 77030, USA; sheeke@mdanderson.org; 7 Department of Oncology, Antoine Lacassagne Center, Université Côte d’Azur, 06100 Nice, France; josiane.otto@nice.unicancer.fr; 8 Department of Thoracic Surgery, Centre Hospitalier Universitaire de Nice, FHU OncoAge, Université Côte d’Azur, 06000 Nice, France; cohen.c@chu-nice.fr (C.C.); gomez-caro-andres.a@chu-nice.fr (A.G.-C.)

**Keywords:** lung, neuroendocrine carcinoma, deep learning, CNN, HALO-AI

## Abstract

**Simple Summary:**

Challenges persist in diagnosing pulmonary neuroendocrine tumors. Our case study shows that deep learning combined with convolutional neural networks has the potential to assist in the diagnosis of pulmonary neuroendocrine tumors from digital whole-slide images.

**Abstract:**

The histological distinction of lung neuroendocrine carcinoma, including small cell lung carcinoma (SCLC), large cell neuroendocrine carcinoma (LCNEC) and atypical carcinoid (AC), can be challenging in some cases, while bearing prognostic and therapeutic significance. To assist pathologists with the differentiation of histologic subtyping, we applied a deep learning classifier equipped with a convolutional neural network (CNN) to recognize lung neuroendocrine neoplasms. Slides of primary lung SCLC, LCNEC and AC were obtained from the Laboratory of Clinical and Experimental Pathology (University Hospital Nice, France). Three thoracic pathologists blindly established gold standard diagnoses. The HALO-AI module (Indica Labs, UK) trained with 18,752 image tiles extracted from 60 slides (SCLC = 20, LCNEC = 20, AC = 20 cases) was then tested on 90 slides (SCLC = 26, LCNEC = 22, AC = 13 and combined SCLC with LCNEC = 4 cases; NSCLC = 25 cases) by F1-score and accuracy. A HALO-AI correct area distribution (AD) cutoff of 50% or more was required to credit the CNN with the correct diagnosis. The tumor maps were false colored and displayed side by side to original hematoxylin and eosin slides with superimposed pathologist annotations. The trained HALO-AI yielded a mean F1-score of 0.99 (95% CI, 0.939–0.999) on the testing set. Our CNN model, providing further larger validation, has the potential to work side by side with the pathologist to accurately differentiate between the different lung neuroendocrine carcinoma in challenging cases.

## 1. Introduction

Pulmonary neuroendocrine tumors (NETs) are a heterogeneous group of neoplasms with variable clinical outcomes [1]. Major pulmonary NET types, such as high-grade and poorly differentiated tumors (e.g., small cell lung carcinoma (SCLC) and large cell neuroendocrine carcinoma (LCNEC)) and low-grade typical and intermediate-grade atypical well-differentiated lung NETs, are included in the World Health Organization (WHO) classification of thoracic tumors and a recent IARC–WHO expert consensus proposal (e.g., typical carcinoid (TC) and atypical carcinoid (AC)) [2,3].

Pulmonary NETs exhibit a wide range of clinical behavior from indolent (typical carcinoids) to rapidly fatal (SCLC) [4]. Pulmonary carcinoids are uncommon malignant tumors that have been growing in yearly occurrence across the world, especially at the advanced stages [5]. Typical carcinoids have a good prognosis with an 80–100% 5-year survival rate, albeit 10–25% of the cases metastasize to regional lymph nodes. Atypical carcinoids have a poorer prognosis, with 40–50% of cases presenting with metastasis, lowering the 5-year overall survival rate to 50% [6]. Unlike pulmonary carcinoids, which can usually be treated with upfront surgery at the time of diagnosis, LCNEC and SCLC require upfront extensive, multimodal treatment for most of the patients. LCNEC patients frequently develop local and systemic metastases, and the cure rate and overall prognosis are dismal, with 5-year survival rates of 13–57% for all patients, 27–62% for early-stage patients and only 5% for late-stage patients [7]. Most patients are diagnosed with extensive disease SCLC and, due to the aggressive behavior of this disease, have a median overall survival between 9 and 12 months [8].

Because of the disparities in clinical therapy and prognosis, it is crucial to make an accurate diagnosis of these tumors. Currently, the most common method in the clinical setting for diagnosing pulmonary NETs is histopathological examination combined with immunohistochemistry (IHC) assessment [9].

The conventional histological criteria for typical carcinoid vs. atypical carcinoid vs. neuroendocrine carcinoma are based on mitotic counts per 2 mm^2^, whereas the presence of necrosis is an additional criterion for atypical carcinoid. Recent reviews summarized the morphologic features of these entities [4,9]. However, there is still no consensus on the most effective approach to make a differential diagnosis, and some diagnostic challenges persist. A surgically resected specimen may usually be used to provide an accurate diagnosis. Nevertheless, approximately 70% of the diagnostic samples are represented by crushed biopsies or cytological samples, which typically lack a well-preserved morphology in the majority of cases, rendering morphological and IHC evaluation difficult [10]. Furthermore, some SCLC and LCNEC borderline subgroups with comparable features make accurate diagnosis challenging [11,12]. Moreover, overall agreement in the diagnosis of lung NETs is achieved in only 70% of cases according to different series [13,14].

As a result, creating a clinically useful complementary tool to identify subtypes of pulmonary NETs more accurately for guiding treatment decisions in routine clinical practice would be of strong value for surgical pathologists.

In recent years, deep learning, in which patterns are learned directly from raw data by convolutional neural networks (CNNs), has achieved remarkable accuracy in image-based recognition and classification [15,16,17]. Deep learning approaches have a great deal of promise for improving histopathological diagnostics by increasing accuracy, reproducibility and speed, as well as reducing the workload of pathologists. Thus, as whole-slide imaging is currently introduced in routine pathology departments, computer-aided diagnosis (CAD) could be a useful tool to improve the interpretation of pathologists. The approach used in our case study showed state-of-the-art results on publicly available histopathology image analysis challenges, namely CAMELYON16 and CAMELYON17, to automate identification and staging of lymph node metastases in breast cancer [18,19]. In thoracic pathology, several prior studies have demonstrated that CNNs can correctly identify morphological patterns and predict survival outcomes or mutation profile using whole-slide images [20,21,22,23]. The CAD was assessed only in distinguishing lung adenocarcinoma from squamous cell carcinoma [20,21,22,23].

Here, we show how the field can further benefit from deep learning by applying a commercialized CNN-based approach to differentiate lung NETs on whole-slide images.

## 2. Materials and Methods

All tumor specimens were used with the informed signed consent from the patients. The study was approved by the local ethics committee (Human Research Ethics Committee, Nice University Hospital Center/hospital-related Biobank BB-0033-00025; http://www.biobank-cotedazur.fr/) and was performed in accordance with the guidelines of the Declaration of Helsinki.

In order to build a classifier that predicts tumor subtypes, highly selected groups of tumors were included in the study. To achieve sample balance, 20 SCLC cases, 20 LCNEC cases and 20 AC cases were selected as the training set (n = 60 cases), whereas 26 SCLC cases, 22 LCNEC cases, 13 AC cases, 4 combined SCLC with LCNEC cases and 25 cases of poorly differentiated adenocarcinoma (negative control) were included in the testing set. The diagnosis routinely made by the expert thoracic pathologists (M.I., V.H., P.H.) has been retained as the gold standard diagnosis for this case selection. Hematoxylin–eosin–saffron (HES) tissue slides were retrieved from the archives of the Laboratory of Clinical and Experimental Pathology, CHU Nice (Nice, France). All the slides originated from patients that underwent surgical resection between 2019 and 2020 (n = 122).

All the slides were scanned with the Nanozoomer 2.0-HT Scanner (Hamamatsu photonics, Hamamatsu, Japan) at ×40 magnification and imported into a computer containing a 12-core, 2.2 GHz Intel Xeon Processor E5-2650 chip and an Nvidia Titan XP graphics card. HALO-AI image analysis software (Indica Labs Inc., London, UK) was used to perform training and testing. The digitized slides were manually annotated by one of three thoracic expert pathologists (M.I., V.H., P.H.). The first class was the designated “tumor” and the second class was “the background” (e.g., stroma, non-tumor).

The HALO-AI train-by-example tissue classification tool is underpinned by 3 advanced deep learning CNNs (VGG, DenseNet and MiniNet). The outcome of the CNNs is subsequently converted into probability maps of SCLC, LCNEC and AC. Training was performed using RMSProp (delta of 0.9) with a learning rate of 1 × 10^3^ reducing the learning rate by 10% every 2k iterations and an L2 regularization of 5 × 10^4^, as previously described [24]. The HALO-AI operator stopped the algorithm once an error rate/cross-entropy rate of less than 0.01 was achieved.

**Figure 1:**
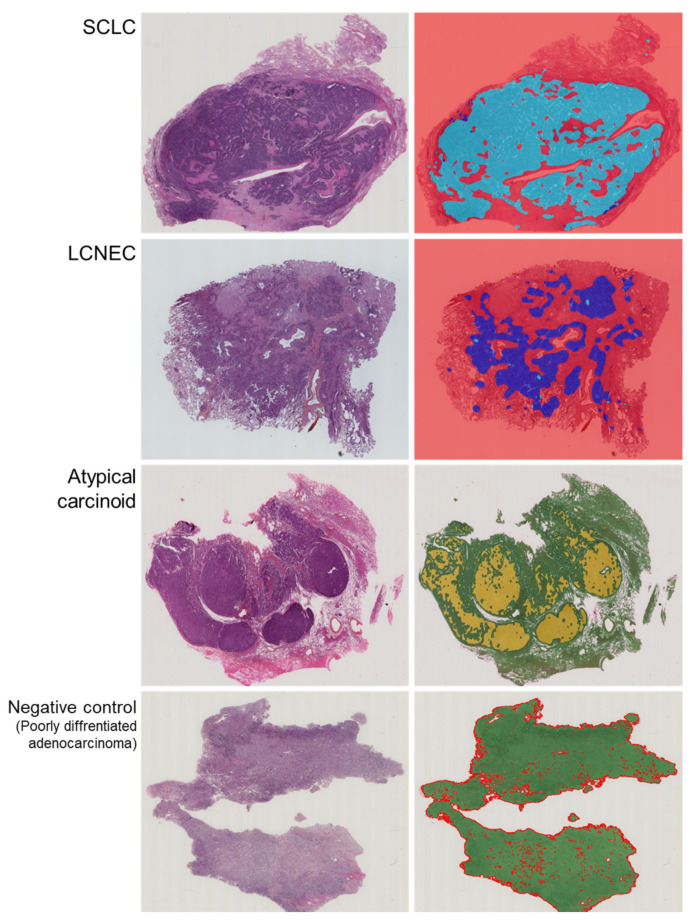
Representative examples of raw images of SCLC, LCNEC, AC and negative cases with an overlap of the HES slide and the corresponding probability maps obtained with the HALO-AI lung NET module trained by the pathologists.

**Figure 2:**
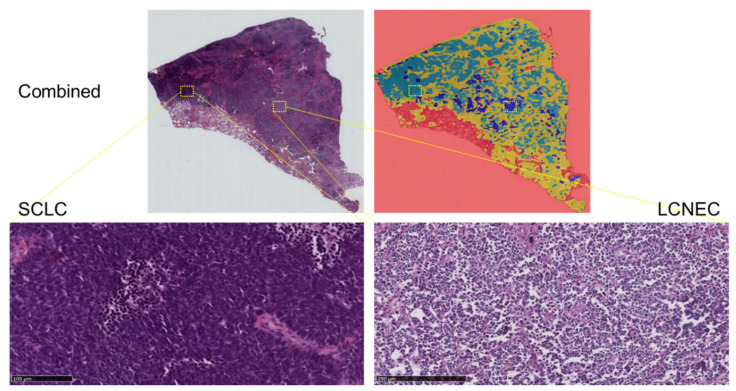
Representative example of raw images of a combined SCLC with LCNEC with an overlap of the HES slide and the corresponding probability maps obtained with the HALO-AI lung NET module trained by the pathologists.

HALO-AI blindly and randomly analyzed the cases included in the testing set, assigning each probability map a likelihood score for that class, which corresponded to the most probable diagnostic call. The output for each test on the whole-slide image was the ratio of the predicted tumor area (in favor of each tumor class) to the total tumor area. This ratio is termed “area distribution” (AD). SCLC was labeled with a light blue label, LCNEC with a dark blue label, AC with a yellow label and the negative control with a green label (Figure 1 and Figure 2).

The receiver operating characteristic (ROC) analysis and the F1-score, accuracy and Cohen’s kappa statistics were used to assess the performance of model predictions. The F-score (F1) considers both precision and recall and is defined as F1 = 2 (precision × recall)/(precision + recall).

## 3. Results

The HALO-AI lung NET module was trained with 18,752 image tiles from the 60 cases in the training set. At an AD percentage cutoff of 50% or more, meaning the HALO-AI module assigned the correct diagnosis to one-half or more of the tissue analyzed, the CNN results were 100% concordant with the gold standard diagnoses for all test sets (e.g., SCLC versus others, LCNEC versus others, AC versus others).

Next, we tested the performance of the HALO-AI lung NET module on the challenging task of distinguishing lung NETs. To assess the accuracy on the testing set, the per-tile classification results were aggregated on a per-slide basis by averaging the probabilities obtained on each tile (mean AD) and compared against the true label, as defined by the pathologist’s gold standard diagnosis.

**Figure 3:**
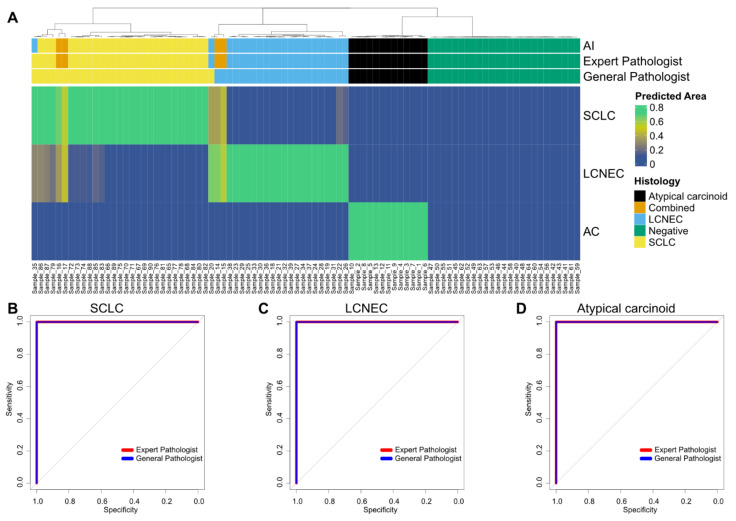
Performance of the HALO-AI lung NET module in the testing set. (**A**) HALO-AI lung NET predicted area distribution calls against gold standard diagnoses. The diagnosis by the AI algorithm, the expert pathologist and the general pathologist is highlighted on top. The heatmap highlights the predicted area of the respective cancer histology (SCLC, LCNEC or AC) as a fraction of 1 (where 1 highlights that the whole area is of the respective histology). A predicted area of greater than 0.5 (50%) was chosen to define the histology based on the AI algorithm. Samples are clustered by Euclidian distance based on the predicted area. (**B**–**D**) ROC analysis for prediction of lung NETs based on the diagnoses of expert and general pathologists. Classification was based on the presence of a histology ((**B**), SCLC; (**C**), LCNEC; (**D**), atypical carcinoid) versus the predicted area as a continuous variable. Analysis for both the expert and the general pathologists is shown. Combined cases were excluded for ROC analysis.

Complete mean HALO-AI AD percentages for the testing set are documented in Figure 3.

For cases of SCLC, the mean AD was 91.93%; for cases of LCNEC, the mean AD was 89.66%; and for cases of AC, the mean AD was 95.72%. Interestingly, two cases of combined SCLC with LCNEC were classified as SCLC (AD = 66.33% and AD = 54.45%), whereas the two remaining cases were classified as LCNEC (AD = 64.15% and AD = 56.16%). On the testing set, the HALO-AI lung NET module yielded a mean F1-score of 0.99, an accuracy of 0.98 (95% CI, 0.937–0.999) and a kappa index of 0.98, demonstrating significant agreement with the gold standard diagnoses (Table 1). No negative controls (poorly differentiated lung adenocarcinoma, n = 25) were recognized by the HALO-AI lung NET module.

We then asked two pathologists (E.L.-M. and S.L.) to independently classify the whole-slide HES images in the test set by visual inspection alone, independently of the classification provided by the HALO-AI module. We measured the agreement between the consensus of each pathologist and of the lung NET module using Cohen’s kappa statistic. The agreement for the gold standard diagnoses of the HALO-AI lung NET module was slightly higher than for the pathologists (0.93 versus 0.84 for pathologist 1 and 0.81 for pathologist 2) but did not reach statistical significance (*p*-values of 0.543 and 0.236, respectively, estimated by a two-sample two-tailed z-test score).

## 4. Discussion

Our case study shows that CNNs, such as HALO-AI, could be used to assist in the diagnosis of pulmonary NETs from histopathology whole-slide images. To our knowledge, this is the first study investigating the use of CNNs to discriminate between several subtypes of pulmonary NETs, which can be quite challenging in some cases. There are borderline neuroendocrine neoplasms that morphologically fall between the different histotypes, and even between expert thoracic pathologists, the agreement can be sometimes hard to achieve.

We trained three CNN algorithms to recognize the major pulmonary NET subtypes, such as high-grade and poorly differentiated tumors SCLC and LCNEC and intermediate-grade atypical well-differentiated lung NETs such as AC.

**Figure 4:**
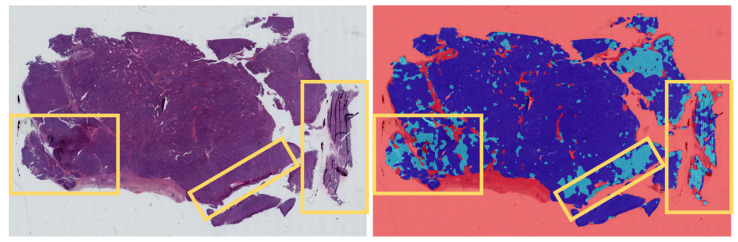
Representative example of raw images of a discordant case of LCNEC as diagnosed by the expert pathologist, while some areas with artifacts (yellow rectangles) were falsely labeled as SCLC by the HALO-AI lung NET module. LCNEC was labeled with a dark blue label, while SCLC was labeled with a light blue label.

The HALO-AI lung NET module distinguishes these subtypes with high accuracy (0.97 F1-score, 0.93 AUC), reaching sensitivity and specificity comparable to those of a pathologist. Interestingly, the few images misclassified by the HALO-AI have also been misclassified by the general pathologists, highlighting the intrinsic difficulty in distinguishing lung NETs in some cases. However, the agreement for the gold standard diagnoses of the HALO-AI lung NET module was slightly higher than that of the pathologists suggesting that the module could be beneficial in assisting the pathologists in their diagnosis. Moreover, the high accuracy of the module was achieved despite the presence of various artifacts in the HES images that were related to the sample preparation and preservation procedures. Nevertheless, these artifacts could explain the two discordant cases observed in our study (Figure 4). This suggests that a better control of tissue slide cutting is needed prior to the scan and analysis by the AI machine.

To improve the differential diagnosis of pulmonary NETs, several approaches have been recently studied combining transcriptomic or IHC-based machine learning algorithms. A prediction model based on the Gene Set Variation Analysis algorithm, enriched with RNA-sequencing data from 13,959 genes, achieved an AUC of 0.949 and a concordance rate of 0.75 for the entire prediction efficiency between SCLC and LCNEC [25]. CNN analysis of established IHC profiles in distinguishing the site of origin of well-differentiated neuroendocrine tumors yielded an agreement of 72% [26]. A deep learning algorithm applied in a pilot study with a limited dataset of archival lung cytological samples of high-grade neuroendocrine carcinoma achieved a mean agreement of 79% in the classification of SCLC versus LCNEC [27]. Recently, a machine learning framework was proposed for better discrimination of the prognosis of lung NETs based on a quantitative, automated and repeatable evaluation of the spatial distribution of cells positive for Ki-67 [28].

In our study, we show that pathologists can further benefit from deep learning algorithms by setting up CNNs on whole-slide images from a wider range of pulmonary NETs, which could be available worldwide. Interestingly, even on complex cases such as combined SCLC with LCNEC, the CNNs were able to achieve accuracy comparable to pathologists.

However, this study has also some limitations. The images used to train or test the CNNs may not fully represent the diversity and heterogeneity of tumors that pathologists typically interpret. More slides and also typical carcinoids would be needed to retrain the CNNs in order to further improve their performance. Moreover, the design of the study is not entirely representative of the full diversity of the “real-life practice” in such heterogeneous tumors. If pathologists had access to IHC and patient information, their accuracy would almost certainly increase. Our algorithm could be further optimized by integrating the Ki-67 index quantification and emerging biomarkers such as RB1, achaete-scute homolog 1-like (ASCL1), neurogenic differentiation factor 1 (NEUROD1) and POU class 2 homeobox 3 (POU2F3) [9,29]. This study is a proof of concept conducted using surgical specimens, therefore with samples of relatively good morphological quality. This approach should be extended to biopsy specimens, both bronchial and transthoracic biopsies, because on small and often crushed specimens the diagnostic difficulties may increase. An independent multicenter validation is necessary before using this approach in daily practice. Finally, independent cohorts were not included because we did not have enough cases to create training and test sets.

## 5. Conclusions

In conclusion, this case study demonstrates that deep learning CNNs could be applied to whole-slide images of lung NETs. Providing further validation, this approach might be a very useful tool for assisting pathologists in their classification of lung NETs. This information could be crucial in choosing the appropriate therapy for patients with lung NETs, thereby increasing the scope and performance of precision medicine. Moreover, when inspecting tumor tissue, pathologists could rely on morphology and may need immunostaining only for the most difficult cases. Although the deep learning analyses may play a role in the initial diagnosis with the benefit of providing important diagnostic information based on an HES image alone, the pathologist has additional tasks, such as staging the tumor and, in an increasing number of cases, estimating response to treatment by the assessment of different predictive biomarkers [30,31].

## Figures and Tables

**Table 1:** Confusion matrix of the prediction of the AI algorithm versus the pathologist (combined cases have been excluded).

	General Pathologist				
		Atypical carcinoid	LCNEC	Negative	SCLC
AI^1^	Atypical carcinoid	13	0	0	0
	LCNEC	0	20	0	2
	Negative	0	0	25	0
	SCLC	0	0	0	26
	**Expert Pathologist**				
		Atypical carcinoid	LCNEC	Negative	SCLC
AI^2^	Atypical carcinoid	13	0	0	0
	LCNEC	0	21	0	1
	Negative	0	0	25	0
	SCLC	0	0	0	26

Accuracy (95% CI): 1 Acc: 0.9767 (0.9185–0.9972); 2 Acc: 0.9884 (0.9369–0.9997).

## Data Availability

Raw data are available upon request.
